# Moderate fabrication and characterization of the microcrystalline Sr_2_CuO_3_ glass films with effective nonlinearities

**DOI:** 10.1038/s41598-018-27967-0

**Published:** 2018-06-22

**Authors:** Tao Xu, Guangcai Hu, Jutao Jiang, Congfei Yin, Run Xiang, Xiaojuan Liang, Weidong Xiang

**Affiliations:** College of Chemistry and Materials Engineering, Wenzhou University, Wenzhou, 325035 China

## Abstract

Since nonlinear optical materials used in the ultrafast all-optical switching is an important part for the modern optical technology, cuprates have been widely investigated for their specific Cu-O chain structure and intriguing optical properties. We present a new preparation method of microcrystalline Sr_2_CuO_3_ glass films on glass substrates combining spin-coating and co-sintering techniques. Then, the as-prepared samples were polished for different times to obtain microcrystalline Sr_2_CuO_3_ glass films with varying thickness. The influence of polishing time on the structure, the valence state and the nonlinear optical response were discussed, respectively. The purity of the Sr_2_CuO_3_ phase, surface morphology and the chemical composites of these synthesized glass films were given with scanning electron microscopy (SEM), energy dispersive X-ray spectroscopy (EDS), X-ray powder diffraction (XRD) and X-ray photoelectron spectroscopy (XPS). Importantly, optical absorption spectroscopy and Z-scan technique were used to measure linear absorption and third-order optical nonlinearity of the films. The experiments showed that third-order nonlinear susceptibility of the 140 min polished film sample with a thickness of 18 μm was up to 1.23 × 10^−12^ esu, indicating its potential application in the nonlinear field.

## Introduction

In recent decades, cuprates with one-dimensional electronic structure have been of particular importance in superconductivity and nonlinear optics due to the extreme CuO_2_ structure and the motion of electrons in a confined space^1–3^. Naturally, Sr_2_CuO_3_, as a typical representative of one-dimensional Mott insulator of cuprates, have been widely investigated for its active role in the preparation of superconductors and unusual nonlinear response^4–8^. In more general terms, the structure of Sr_2_CuO_3_ is similar to that of the two-dimensional compound La_2_CuO_4_, which only lacks oxygen atoms in Cu-O chains between the Cu ions in the c-axis direction, therefore, Sr_2_CuO_3_ is a favorable precursor for the synthesis of Cu-O composites^9–12^. Previous studies focus much on searching for the analogs of Sr_2_CuO_3_ or introducing other metallic elements to enhance the superconductivity of the final product. For example, Koushik Karmakar prepared single crystal SrCuO_2_ using the Traveling Solvent Floating Zone (TSFZ) Method and studied its magnetic property^13^. After that, Koushik Karmakar successfully achieved crystal growth of spin chain compound Sr_2_CuO_3_ doped with Zn, Co, Ni and Mn and analyzed quantum defect in a spin 1/2 chain^14^. Additionally, T. Geballe synthesized the Sr_2−x_Ba_x_CuO_3+δ_ through a traditional solid state method, mainly to enhance the superconductivity of the cuprates with the doping of Ba atoms^15^. The used Sr_2_CuO_3_ powder is mostly synthesized through the traditional solid state method, and the electronic structure and superconductivity of the related materials are investigated^16–20^. However, these studies had been devoted to understand the structure and size of Sr_2_CuO_3_ particles, lacking the exploration in nonlinear applications field. Fortunately, the Sr_2_CuO_3_ with large third-order nonlinearity had also been reported in 2000. H. Kishida studied the third-order nonlinearity of one-dimensional Mott insulators of Sr_2_CuO_3_ thin film and analyzed the nonlinear enhancement mechanism for the first time^21^. Additionally, T. Ogasawara reported high third-order susceptibility χ^(3)^ at optical fiber communication and room temperature ultrafast recovery on Sr_2_CuO_3_ thin film, indicating the strong potential of Sr_2_CuO_3_ to fabrication of all-optical switching devices^22^. Besides that, H. Kishida obtained third-harmonic generation spectra in single-crystalline thin films of Sr_2_CuO_3_ and Ca_2_CuO_3_^23^. The two-photon resonant structure demonstrates that the even-charge transfer states are located close to the odd-charge transfer states. After that, M. Ohtani revealed the synthesis of desired orientations and high crystallinity for (Sr_x_Ca_1−x_)_2_CuO_3_ thin films and the linear relation of x and the charge transfer gap^24^. Furthermore, A. Maeda had successfully grown Sr_2_CuO_3_ crystals on the LaAlO_3_ substrate, and then discussed its nonlinear property with Ca_2_CuO_3_ to considering excitonic effect^25^. In a word, scholars prepare Sr_2_CuO_3_ materials with the use of pulsed laser deposition (PLD), TFSZ and RF magnetron sputtering techniques, which are always labor-intensive and time-consuming with the instrument hard to manipulate. Furthermore, the research of the thickness dependence of structure and optical property for the microcrystalline Sr_2_CuO_3_ films is seldom reported.

In this paper, microcrystalline Sr_2_CuO_3_ glass films were obtained by combining co-sintering and spin-coating technique for the first time^26,27^. The Sr_2_CuO_3_ particles were firmly embedded in the Pb-glass matrix on the surface of K_9_ glass substrate. Subsequently, through mechanical polishing for different times, different thicknesses of film samples were acquired. Thickness dependence of structure and optical property for the microcrystalline Sr_2_CuO_3_ glass films were investigated in detail. As a result, the final Z-scan experiment showed that the 140 min polished microcrystalline Sr_2_CuO_3_ glass film with a thickness of 18 μm owned a fast nonlinear response, could be a suitable nonlinear optical candidate compared to some known materials^28^.

## Results and Discussion

### SEM and EDS analysis

**Figure 1:**
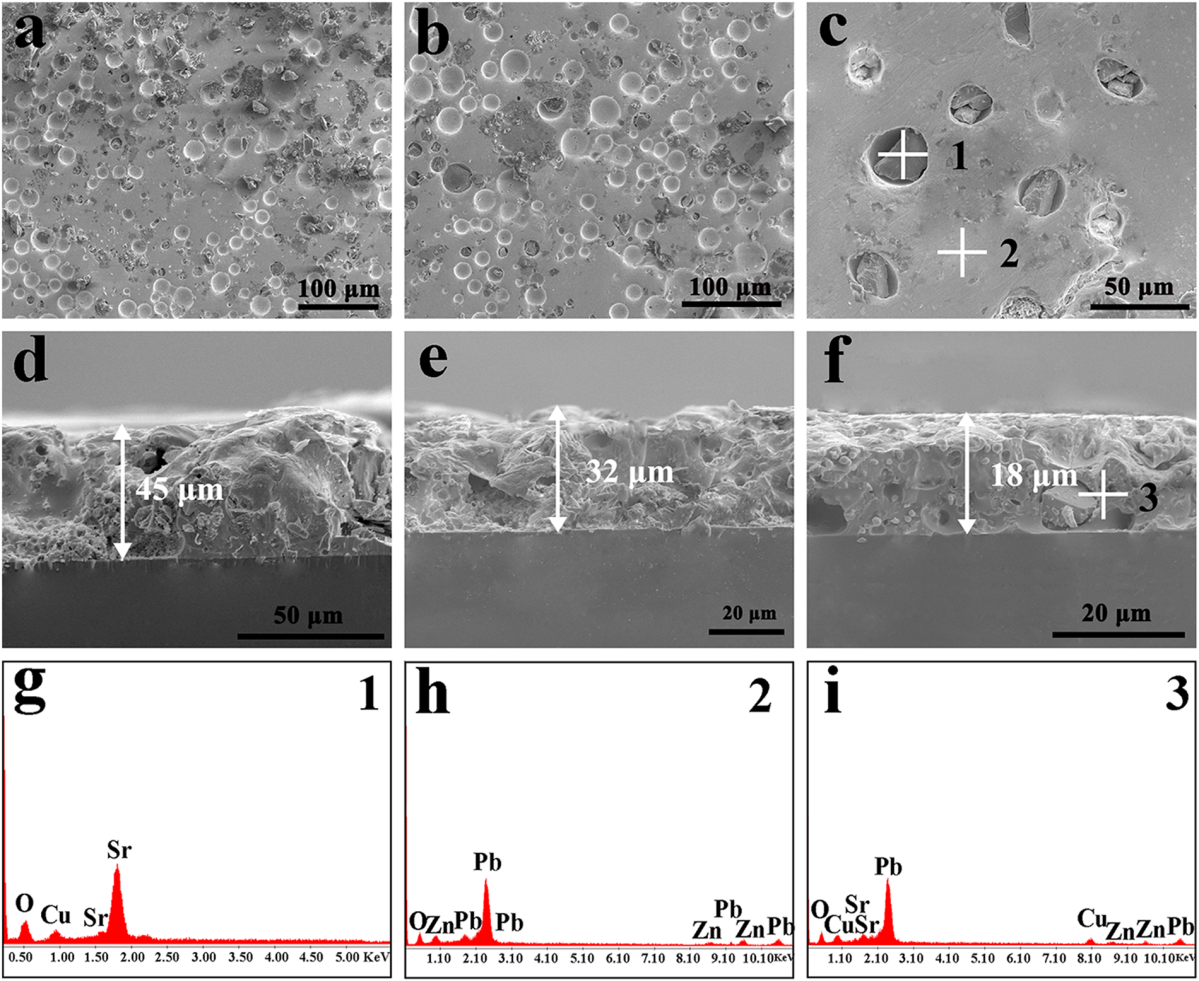
Morphology and structural characterization of the microcrystalline Sr_2_CuO_3_ glass films polished different times under 500 °C sintering: (**a**)/(**d**) 60 min, (**b**)/(**e**) 100 min and (**c**)/(**f**) 140 min. The EDS images of the microcrystalline Sr_2_CuO_3_ glass film polished for 140 min at three points (**g**) 1, (**h**) 2 and (**i**) 3.

To capture the transformation of the morphology and elements distribution in the microcrystalline Sr_2_CuO_3_ glass film under the polishing process, SEM and EDS spectra analyses were conducted. Figure 1a–c provide the SEM images of the microcrystalline Sr_2_CuO_3_ glass film at three polishing times: 60, 100 and 140 min under 500 °C sintering, as an example. The as-prepared films reveal that the gray microcrystalline Sr_2_CuO_3_ particles were tightly embedded in the Pb-glass layer, which were fabricated by using co-sintering the original materials, and they were adhered to the surfaces in the K_9_ glass substrates. With the increase of polishing times, the surfaces of microcrystalline glass film transformed from rough and holey morphology to relatively well-distributed and smooth surfaces. As though there are still some sporadic holes exist in the cross-sections of the various film sample, and the smooth skin layers illustrate the polishing process could effectively adjust the film thickness, as shown in Fig. 1d–f. Additionally, by measurement, the corresponding thicknesses of these film samples polished for 60 min, 100 min, 140 min were 45, 32 and 18 μm, respectively. The unique structure and steerable thickness of the as-synthesized film samples have a large impact on the valence state and the nonlinear optical response, which will be discussed below. Furthermore, the EDS spectra of these film samples are presented in Fig. 1g–i, revealing there are no impurities in these films except the Sr_2_CuO_3_ particles and the Pb-glass matrix.

### XRD analysis

**Figure 2:**
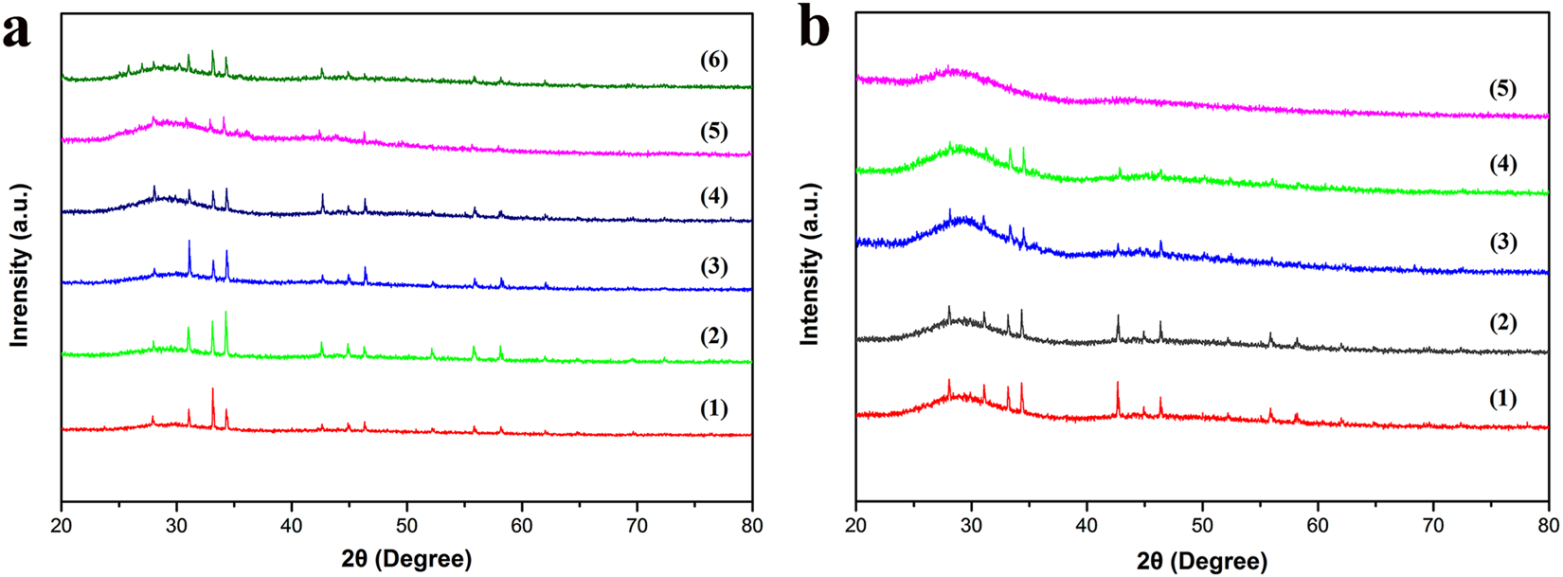
(**a**) Comparison of powder XRD patterns of the microcrystalline Sr_2_CuO_3_ glass films with different sintering temperatures (1) 100 °C, (2) 200 °C, (3) 300 °C, (4) 400 °C, (5) 500 °C and (6) 600 °C. (**b**) Comparison of powder XRD patterns of the microcrystalline Sr_2_CuO_3_ glass films polished different times under 500 °C sintering (1) 0 min, (2) 60 min, (3) 100 min, (4) 140 min and (5) 180 min.

To assess the influence of the dependence of synthesis temperature and polishing time on the structure of microcrystalline Sr_2_CuO_3_ glass film, the XRD patterns are presented in Fig. 2. The crystalline and purity of microcrystalline Sr_2_CuO_3_ glass film sintered at 100 °C to 600 °C orderly may be depicted as in Fig. 2a. Obviously, the diffraction patterns correspond to the JCPDS 85–2487 (Sr_2_CuO_3_) from 100 °C to 500 °C. No extra peaks are detected, which indicate that the Sr_2_CuO_3_ has a single-phase structure during the whole heating process. Nevertheless, there are some extra peaks conforming to PbO and SrPbO_3_ appear when the sinter temperature rise up to 600 °C, mainly attribute to the interaction between Sr_2_CuO_3_ and the glass compositions. In addition, Fig. 2b shows the effect of different polishing times vary from 0 to 180 min on the particle size of microcrystalline film prepared at 500 °C, considering the better combination of glass substrate and Sr_2_CuO_3_ particles and phase purity. We can see that as the polishing time increases, the intensity of the diffraction peak gradually decreases. From the XRD patterns of the microcrystalline Sr_2_CuO_3_ glass film polished for 180 min, we could assume that the surface of the film sample is K_9_ glass substrate. Similarly, all the XRD patterns exhibit a large amorphous peak with 2θ at approximately 28°~32° attributing to the diffraction from the glass matrix.

### XPS analysis

**Figure 3:**
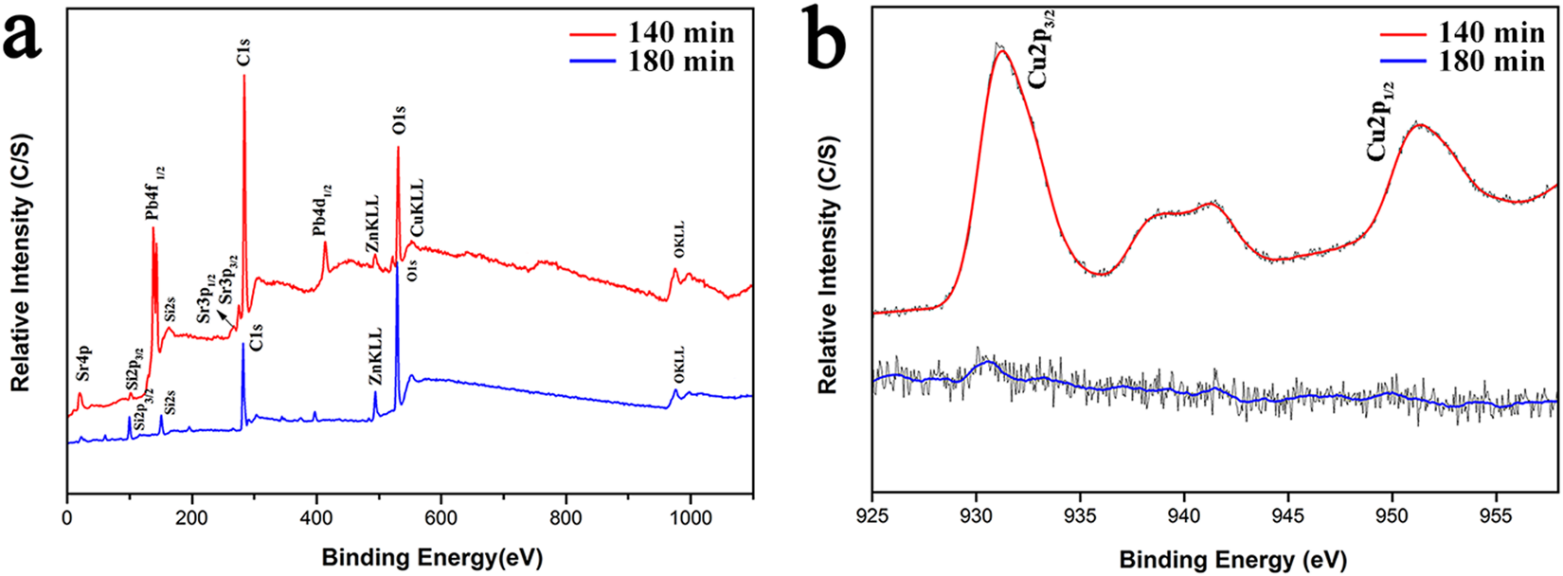
XPS spectra of the film samples polished 140 min and 180 min under 500 °C sintering: (**a**) full XPS spectra and (**b**) the high-resolution spectra for Cu 2p.

With the purpose of evaluating the effect of the thermal treatment and polish procedure on the valence state and element type of the microcrystalline Sr_2_CuO_3_ glass films, ulteriorly, the XPS was performed to analyze the as-prepared samples polished for 140 min and 180 min under sintering temperature of 500 °C. Figure 3a depicts the survey XPS spectra of these two glass films correspond to the red and blue curves for the samples polished for 140 min and 180 min, respectively. From the red curve, we can observe that the peaks of Pb, Zn, Si, Sr, Cu and O were presented in the as-synthesized glass film, agreeing with glass composition and Sr_2_CuO_3_. From XPS and fitting curves, we can calculate that the atomic ratio of Sr and Cu was 2:1, which correspond to the theoretical value (Sr_2_CuO_3_ particles). Meanwhile, the film sample polished for 180 min was also tested, mainly contain Zn, Si, and O from the blue curve, indicating that the film sample was in accordance with the K_9_ glass substrate under excessive polishing procedure and no external element was introduced. Additionally, the high-resolution spectra for Cu 2p in these two film samples polished for 140 min and 180 min were showed in Fig. 3b. The red curve gives two peaks of Cu 2p_3/2_ and Cu 2p_1/2_ at approximately 931.9 eV and 951.16 eV, respectively, illustrating the valence state of Cu in the film sample is +2 or +1^28^. As for the other, the Cu signals don’t appear on the blue curve. Obviously, the red curve in Fig. 3b shows a precisely characteristic peak of Cu^2+^ at about 942.16 eV between the two strong peaks conforming to the XRD analysis of the 140 min polished microcrystalline Sr_2_CuO_3_ glass film.

### Third-order optical nonlinearity

**Figure 4:**
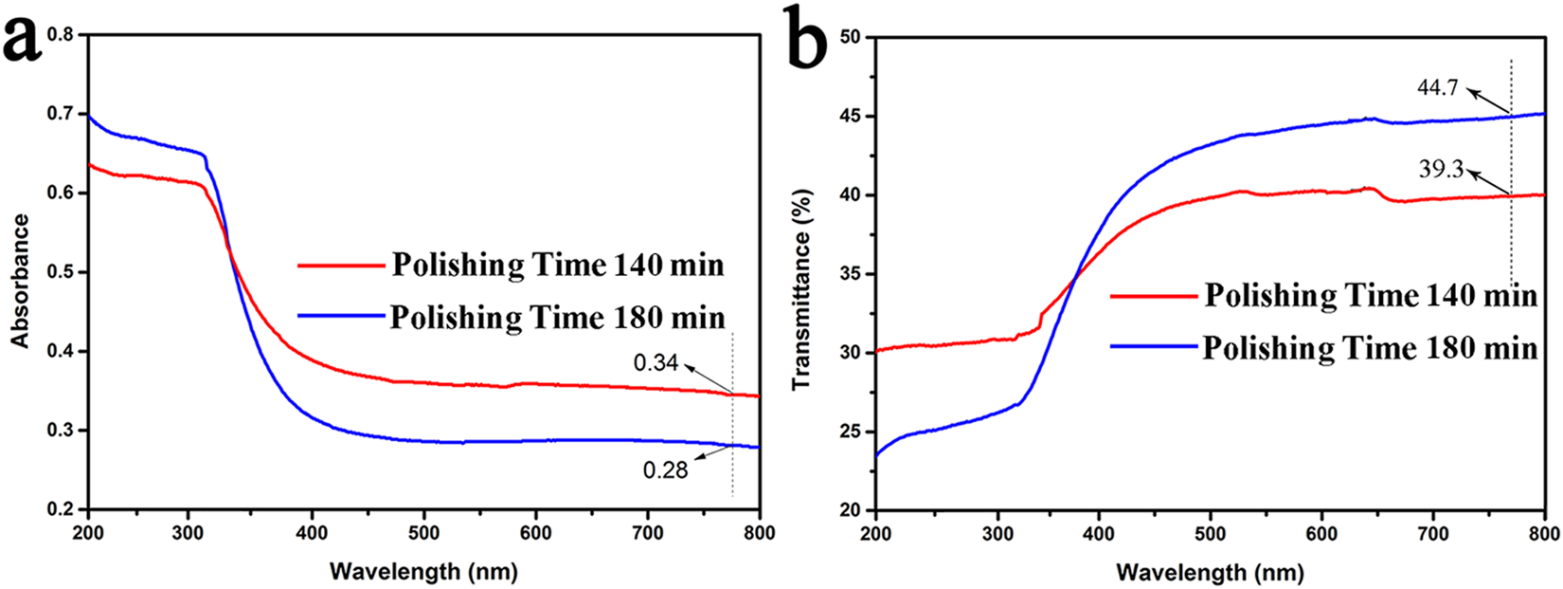
UV/VIS spectra of the film samples polished 140 min and 180 min: (**a**) Linear optical absorption spectra and (**b**) transmittance spectra.

Optical absorption spectroscopy and Z-scan technique were used to measure linear absorption and third-order optical nonlinearity of the microcrystalline Sr_2_CuO_3_ glass films. Linear optical absorption of the film samples polished for 140 min and 180 min are described in Fig. 4a. In general, while for the sample with a lower transmittance (less than 30%), most of the testing energy are absorbed by the sample in the process, causing an increase of thermal effects on the sample. The linear optical absorption includes the linear loss. Figure 4b reveals that the transmittance of the glass film is about 40% at a wavelength of 787 nm, meeting the essential requirements of the Z-scan measurements. While the other glass films polished for shorter time would gather high powers on the surface under excitation laser irradiation, affecting the accuracy of measurement seriously for the lower transmittance.

**Figure 5:**
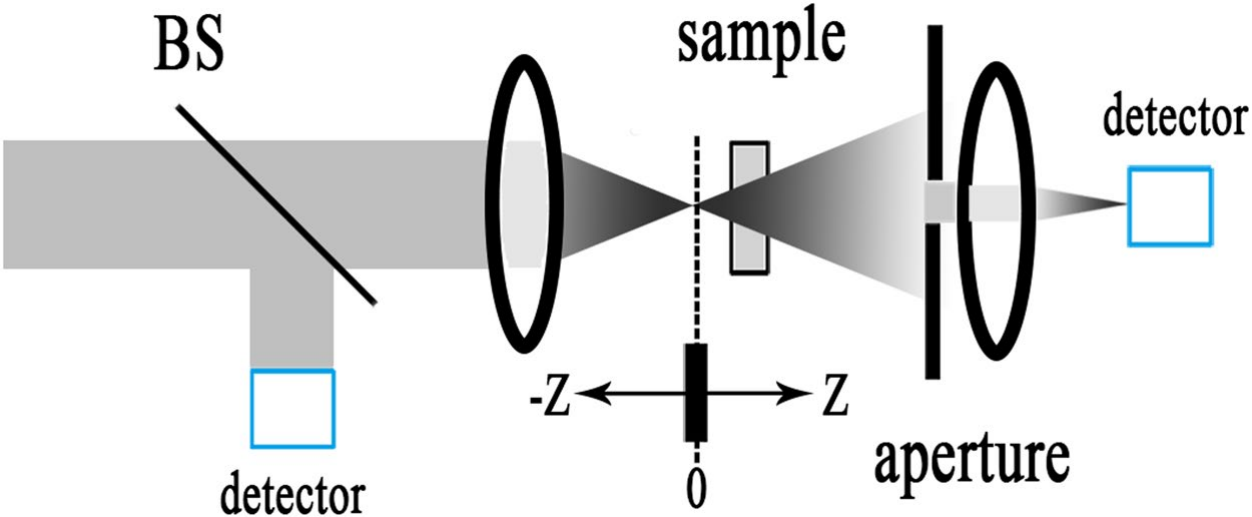
Schematic diagram of the OA (**a**) and CA (**b**) *Z*-scan experiment.

**Figure 6:**
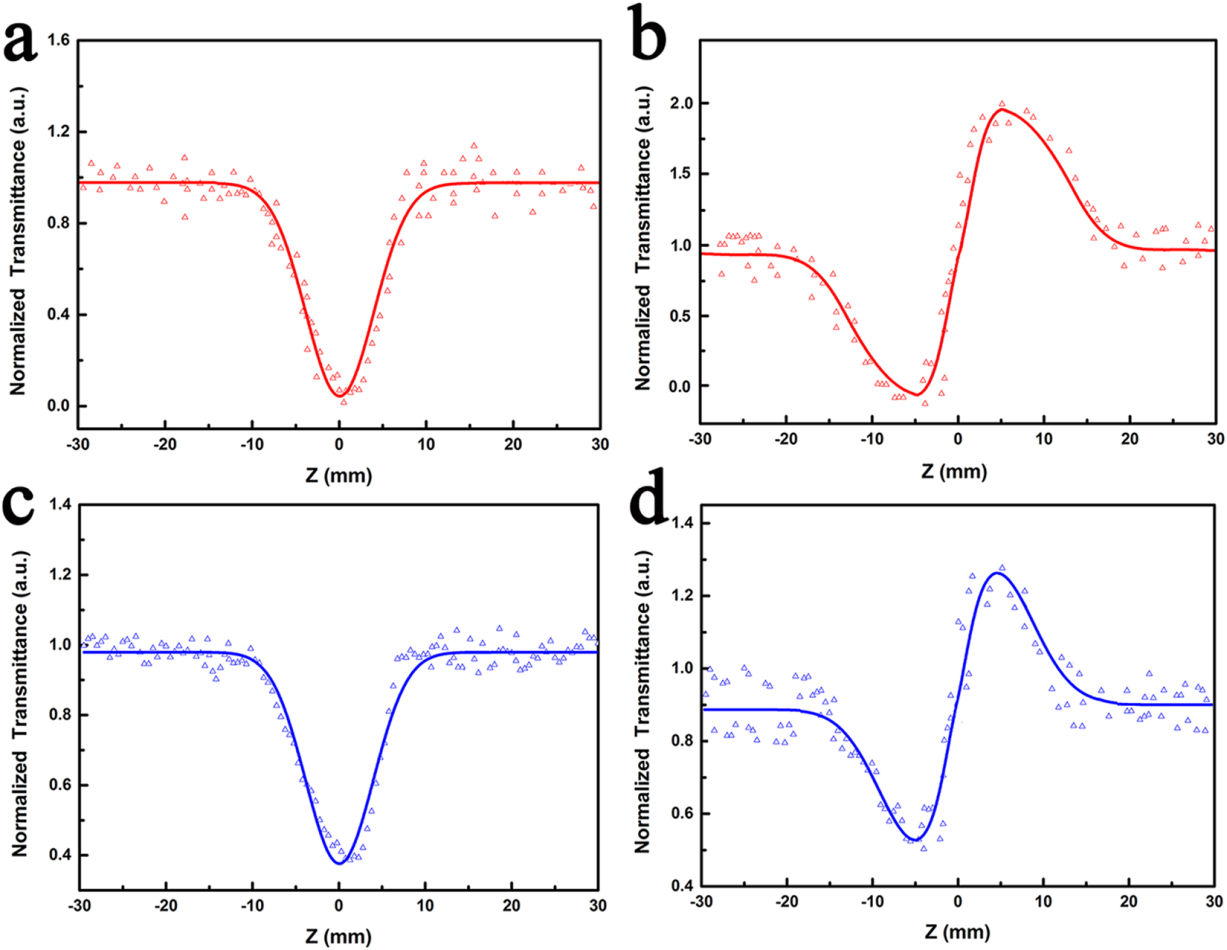
OA signal (**a**) and CA signal (**b**) of the film sample polished for 140 min. OA signal (**c**) and CA signal (**d**) of the film sample polished for 180 min.

The Z-scan is a single excitation beam technique to measure the nonlinear refractive index and nonlinear absorption coefficient of the materials^29^. As can be easily understood, Fig. 5 simply shows schematic diagram of the Open-aperture (OA) and closed-aperture (CA) Z-scan experiment. The nonlinear refraction and absorption of the microcrystalline Sr_2_CuO_3_ glass films polished for 140 and 180 min were acquired by using OA and CA Z-scan technology. As shown in Fig. 6, the hollow triangles represent the measure results and the solid line show the theoretical fit of the normalized transmittance. Figure 6a and b give the OA and CA Z-scan results for the microcrystalline Sr_2_CuO_3_ glass film polished for 140 min. The film sample polished for 180 min was contrasted for reference shown in Fig. 6c,d. We can see that this is the reverse saturable absorption in the microcrystalline Sr_2_CuO_3_ glass film on account of the single symmetrical valley of peak relative to the focus (z = 0) of the solid line in Fig. 6a. Meanwhile, Fig. 6b displays the signature of valley of peak indicating the occurrence of self-focusing process and negative value for the nonlinear refractive index (γ < 0) in the film sample at CA measurements. As well for the glass substrate in Fig. 6c,d, the signature is similar to the film sample polished for 140 min but the intensity is relatively weak.

According to a well-known theory^30^, from the OA and CA Z-scan curves, we can calculate the nonlinear absorption coefficient (β) and nonlinear refractive index (γ) of the film. Furthermore, we can calculate the real and imaginary parts of the third-order nonlinear susceptibility (χ^(3)^) of the film samples by equations (1) and (2)^31^,

1 $${\mathrm{Im}{\rm{\chi }}}^{(3)}(\mathrm{esu})=\,\frac{{{\rm{\lambda }}{\rm{\varepsilon }}}_{{\rm{0}}}{{\rm{c}}}^{{\rm{2}}}{{{\rm{n}}}_{{\rm{0}}}}^{{\rm{2}}}}{{4{\rm{\pi }}}^{{\rm{2}}}}\beta ({\rm{m}}{\rm{/}}{\rm{W}})$$

2 $${\mathrm{Re}{\rm{\chi }}}^{(3)}(\mathrm{esu})=\,\frac{{{\rm{\varepsilon }}}_{{\rm{0}}}{{\rm{c}}}^{{\rm{2}}}{{{\rm{n}}}_{{\rm{0}}}}^{{\rm{2}}}}{\,{\rm{\pi }}}\gamma {({\rm{m}}}^{{\rm{2}}}{\rm{/}}{\rm{W}})$$

where n_0_, c, ε_0_ and λ are linear refractive index, speed of light, permittivity of free space and the wavelength of the laser light of the film sample, respectively. The total third-order nonlinear susceptibility χ^(3)^ can be calculated by equation (3).

3 $${|{\rm{\chi }}}^{(3)}{|(\mathrm{esu})=\{(\mathrm{Re}{\rm{\chi }}}^{(3)}{)}^{{\rm{2}}}{+(\mathrm{Im}{\rm{\chi }}}^{(3)}{)}^{{\rm{2}}}{\}}^{1/2}$$

As shown in Table 1, the parameters of third-order optical nonlinearity are listed. We introduced microcrystalline Sr_2_CuO_3_ in the Pb-glass matrix on the surface of K_9_ glass substrate, and the results were expected to lead to a large third-order nonlinearity of the film sample, whose nonlinear susceptibility could be as high as 1.23 × 10^−12^ esu. Furthermore, we introduce the photon theory to explain the third-order nonlinear phenomenon more deeply. In the one-dimensional Cu-O composites, due to the occupied p-band of oxygen between the upper and lower Hubbard bands of the metal and the big field Coulomb interactions at the metal sites, the Mott-Hubbard gap opens in the d-band of copper^21^. The charge transfer between Cu and O is major determinant of the nonlinear optical response, while the Sr only serves as a bridge which concatenates the parallel Cu-O chains. In short, the absorption of photons and the dipole moment in the Cu-O composites result in the noticeably increase of third-order nonlinear susceptibility χ^(3)^ principally.

**Table 1:** Third-Order Nonlinear Optical Parameters of the film samples polished for 140 min and 180 min.

Parameters	β(m/w)	Imχ^(3)^(esu)	γ (m^2^/w)	Reχ^(3)^(esu)	χ^(3)^(esu)
Microcrystalline Sr_2_CuO_3_ film (140 min)	1.4 × 10^−11^	4.69 × 10^−13^	2.1 × 10^−18^	1.14 × 10^−12^	1.23 × 10^−12^
Microcrystalline Sr_2_CuO_3_ film (180 min)	3.7 × 10^−13^	1.35 × 10^−14^	8.9 × 10^−20^	4.82 × 10^−14^	5.0 × 10^−14^

## Conclusions

In a word, we report the synthesis of microcrystalline Sr_2_CuO_3_ glass films, which were fabricated by co-sintering and spin-coating technique. Next, through continuous polishing, we acquire microcrystalline films with various thicknesses. The changes of microcrystalline Sr_2_CuO_3_ glass films during the polishing process were monitored and discussed individually. From the analysis results of the SEM and EDS spectra, the Sr_2_CuO_3_ crystalline phase were well-embedded in the glass matrix. Additionally, the XRD patterns of the polished film samples exactly verified that the existence of Sr_2_CuO_3_ microcrystals in the glass matrices. At the same time, the composition and binding energy of Sr, Cu, and O were determined using the results of XPS curves. From XPS and calculations, we can concluded that the atomic ratio of Sr and Cu was 2:1, which correspond to the theoretical value (Sr_2_CuO_3_ particles). Benefiting from the results, third-order optical nonlinearities of the microcrystalline Sr_2_CuO_3_ glass films were measured through the Z-scan technique. The Z-scan experiment indicated that the microcrystalline Sr_2_CuO_3_ glass film has a terrific third-order optical nonlinearity with the χ^(3)^ as high as 1.23 × 10^−12^ esu, exhibiting its potential application in the nonlinear field.

## Methods

### Samples preparation

The Sr_2_CuO_3_ powder was prepared to use a traditional solid state method. SrO (99.99%) and CuO (99.99%) in stoichiometric amount were mixed evenly for 60 min, and the mixture was sintered at 980 °C for 20 h in the crucibles under an ambient atmosphere. After a short time, the subsequent acquired intermediates were again further heated at 980 °C for 10 h, with fully grindings. Subsequently, the Sr_2_CuO_3_ powder in grayish was obtained. It is pointed out that the synthesized Sr_2_CuO_3_ powder is hygroscopic, which should be stored in the dryer to avoid contact with moist air and carbon dioxide. After that, according to the ratio of 1:3 (mass fraction), the Sr_2_CuO_3_ and Pb-glass powder (PbO_2_-ZnO-B_2_O_3_-SiO_2_) were mixed homogeneously for 2 h. Then, their mixtures were blended with the naphtha evenly. The viscous composite was individually disposed on the surfaces of K_9_ glass substrate through spin-coating technology with the rate of 2000 rpm for 30 s. Subsequently, we put the composites of Sr_2_CuO_3_ and glass powder on the K_9_ glass substrates into the muffle furnace, and adjust the sintering temperature from 100 °C to 600 °C. Last, we acquired film samples, which were named microcrystalline Sr_2_CuO_3_ glass films by us, and the as-prepared samples were polished for different times to obtain microcrystalline Sr_2_CuO_3_ glass films with varying thickness.

### Samples tests

All of the film samples, no matter what they prepared in any conditions, were subjected to structural and phase characterization with the X-ray diffraction (XRD, D8 Focus, Bruker, Germany) in the range of 20–80^o^ (2θ). In addition, the morphology and elements distribution in the microcrystalline Sr_2_CuO_3_ glass film were analyzed by field-emission scanning electron microscope (FE-SEM, Auriga, Carl Zeiss) with the measuring equipment of energy dispersive X-ray spectroscopy. The chemical compositions and the valence state of ions in the thin film surface were characterized with the X-ray photoelectron spectroscopy (XPS, SHI-MADZU) Furthermore, by using the UV*/*VIS Spectrophotometer (Mode V-570, JASCO), the linear absorption and transmittance spectra were acquired. Measurements of the third-order optical nonlinearities of the microcrystalline Sr_2_CuO_3_ glass film were determined by Z-scan method at 787 nm, and the pulse width, single-pulse energy and repetition rate were 397 fs, 0.1 μJ and 1000 Hz, respectively. All of the above tests of the film samples were conducted at room temperature.

### Data availability

The datasets generated during the current study are available from the corresponding author on reasonable request.
